# Effects of Phenolic‐Rich Extra Virgin Olive Oil and Prebiotics on Sarcopenia in Older Adults: FOOP‐Sarc Project

**DOI:** 10.1002/jcsm.70247

**Published:** 2026-03-05

**Authors:** Maria Besora‐Moreno, Claudia Jiménez‐ten Hoevel, Judit Queral, Glòria Bernal, Laura Pérez‐Merino, José Puzo, Laura Conangla‐Ferrin, Leann Constance Sleebos, Wout van Helden, Elisabet Llauradó, Rosa M. Valls, Rosa Solà, Anna Pedret

**Affiliations:** ^1^ Facultat de Medicina i Ciències de la Salut, Functional Nutrition, Oxidation, and Cardiovascular Diseases Group (NFOC‐Salut) Universitat Rovira i Virgili Reus Spain; ^2^ Hospital Universitari Sant Joan de Reus Reus Spain; ^3^ Hospital Universitario San Jorge Huesca Spain; ^4^ EAP Badalona Centre i Dalt La Vila, Institut Català de la Salut Badalona Spain; ^5^ Grup de Recerca ecoAP IDIAP Jordi Gol i Gurina Barcelona Spain; ^6^ Grup de Treball d'Ecografia en Atenció Primària (ecoAP) de CAMFiC Barcelona Spain; ^7^ Natura Foundation Numansdorp the Netherlands; ^8^ Institut d'Investigació Sanitària Pere i Virgili (IISPV) Reus Spain

**Keywords:** isokinetic, older adults, olive oil, prebiotic, sarcopenia, ultrasound

## Abstract

**Background:**

Management of sarcopenia by nutritional and lifestyle interventions is a challenge. The aim is to assess the effectiveness of a phenolic‐rich extra virgin olive oil (EVOO), alone or combined with a prebiotic (PREB) (fructooligosaccharides and inulin), to improve skeletal muscle mass and function in home‐dwelling older adults (60–80 years) with at least one sarcopenia parameter altered.

**Methods:**

A 12‐week randomised, double‐blind, parallel, placebo‐controlled, three‐arm clinical trial was conducted. Intervention groups were as follows: (1) refined olive oil (ROO; 30 mL/day; 90‐mg caffeic acid) + maltodextrin placebo (7.5 g/day); (2) EVOO (30 mL/day; 296–300‐mg caffeic acid) + maltodextrin placebo (7.5 g/day); or (3) EVOO + prebiotic (EVOO + PREB; 30 mL/day + 7.5 g/day). Everyone followed co‐created dietary and physical activity recommendations. A 12‐week follow‐up after intervention cessation was assessed.

**Results:**

Thirty‐eight participants (69.6 ± 4.1 years; 31 women) with probable sarcopenia were assigned to ROO (*n* = 13), EVOO (*n* = 14) or EVOO + PREB (*n* = 11) intervention groups. At the end‐of‐intervention, assessed by ultrasound, EVOO + PREB compared to EVOO significantly increased muscle thickness of the quadriceps in females, mean (95% CI) (0.230 cm [0.008; 0.45], *p* = 0.044), cross‐sectional area of the rectus femoris in all populations (0.827 cm^2^ [0.16; 1.5], *p* = 0.017) and females (0.569 cm^2^ [−1.0; −0.08], *p* = 0.024), and rectus femoris muscle thickness in all population (0.195 cm [0.04; 0.35] *p* = 0.015) and females (0.179 cm [0.05; 0.31] *p* = 0.009). Also, EVOO + PREB compared to ROO increased the cross‐sectional area of the rectus femoris (0.579 cm^2^ [0.07; 1.1], *p* = 0.026) and rectus femoris muscle thickness (0.133 cm [0.00; 0.27], *p* = 0.050) in females. At 12‐week follow‐up, EVOO and EVOO + PREB, compared to ROO, significantly increased skeletal muscle mass and appendicular skeletal muscle mass in all populations assessed by bioelectrical impedance analysis (BIA). Also, EVOO, compared to ROO, significantly increased skeletal muscle mass index and appendicular skeletal muscle mass index. In addition, at 12‐week follow‐up, EVOO, compared to ROO, improved the overall quality of life score in females.

**Conclusions:**

Consuming phenolic‐rich EVOO, alone or combined with prebiotics, improved muscle mass assessed by ultrasound at the end‐of‐intervention and by BIA at 12‐week follow‐up. Further studies are needed to confirm these promising results.

**Trial Registration:** Registration number: NCT05485402; https://clinicaltrials.gov/study/NCT05485402; registration date: 03/08/2022.

## 1 Introduction

Sarcopenia is a decline in skeletal muscle mass (SM) and function related to ageing [1]. Nowadays, the number of older people over 60 is increasing worldwide [2] and sarcopenia prevalence in Europe is projected to increase from 11.1% in 2016 to 22.3% in 2045 [3]. Sarcopenia consequences are high risk of fractures and falls, loss of independence, mortality and increasing hospitalisations and healthcare costs [4, 5].

As reported by the European Working Group on Sarcopenia in Older People (EWGSOP2, 2019), sarcopenia is defined based on three key parameters: muscle strength, muscle mass and physical performance [1]. According to EWGSOP2 classification, low muscle strength indicates probable sarcopenia; confirmation requires both low muscle strength and low muscle mass, while the addition of poor physical performance indicates severe sarcopenia [1]. In comparison of EWGSOP2, the earlier EWGSOP1 (2010) consensus, with different cut‐off points, is based on muscle mass as the first parameter for the diagnosis of sarcopenia rather than muscle strength [6].

To evaluate muscle mass, ultrasound is an emerging, cheap, portable and non‐invasive technique, with advantages over gold standard techniques such as computed tomography (CT), magnetic resonance imaging (MRI) or dual photon X‐ray absorptiometry (DXA) that are less accessible in clinical practice and more expensive [7]. Commonly, ultrasound is used in larger muscle groups such as the quadriceps [8]. Muscle thickness (MT) and cross‐sectional area (CSA) have been strongly correlated with muscle mass gold standard techniques [7]. Additionally, bioelectrical impedance analysis (BIA) of muscle mass is more affordable and portable than DXA, but validated prediction equations for specific populations are necessary [1].

The underlying mechanism of sarcopenia is still being investigated, but there is evidence that oxidative stress and inflammation are key contributing factors [9]. The excessive production of reactive oxygen species (ROS) contributes to oxidative damage, loss of muscle mass, function and contractility deterioration [9, 10, 11]. Related to nutritional management of sarcopenia, extra virgin olive oil (EVOO) seems to reduce the symptomatology of sarcopenia, reducing oxidative damage produced by ROS and inflammation [10, 11]. Furthermore, prebiotics—a substrate used selectively by the microorganisms of the host to confer health benefits [12]—may represent a novel therapeutic approach via the gut‐muscle axis [13]. Previous studies have shown that prebiotic supplementation with fructooligosaccharides (FOS) and inulin in older adults (≥ 65 years) can improve frailty [14]. Frailty is a condition that included five criteria: unintentional weight loss, weakness based on grip strength, poor endurance and energy, slowness and low physical activity level [15]. Nevertheless, the potential impact of these prebiotics on muscle mass, which is a specific parameter that define sarcopenia, remains unknown. Besides, following a diet rich in protein, leucine, vitamin D and antioxidant nutrients, together with performing at least 150 min/week of moderate‐to‐vigorous physical activity, can help prevent sarcopenia by countering the loss of muscle mass [16].

Thus, nutritional and lifestyle interventions remain a challenge in sarcopenia management, and the combination of EVOO and prebiotics requires further investigation, as well as exploring whether the effects of an intervention are detected after the intervention cessation.

The FOOP‐Sarc Project hypothesises that a dietary strategy based on an EVOO rich in phenolic compounds alone or in combination with prebiotic supplementation (FOS and inulin) will significantly improve SM and function in home‐dwelling older adults (60–80 years) with at least one sarcopenia parameter altered.

The main objective of the present study is to assess the effectiveness of a dietary strategy based on an EVOO rich in phenolic compounds alone or in combination with prebiotic supplementation (FOS and inulin) to improve SM and function in home‐dwelling older adults (60–80 years) with at least one sarcopenia parameter altered.

## 2 Methods

### 2.1 Study Design and Intervention

A 24‐week randomised, double‐blind, parallel, placebo‐controlled, three‐arm clinical trial was conducted between September 2022 and July 2023 in Reus and its outskirts (Spain). The clinical trial was structured into 12 weeks of intervention and 12 weeks of follow‐up after intervention cessation. The intervention products were as follows: (a) EVOO rich in phenolic compounds (156‐mg hydroxytyrosol and tyrosol/kg oil and 296–300‐mg caffeic acid as total polyphenols) or refined olive oil (ROO) (38.6‐mg hydroxytyrosol and tyrosol/kg oil and 90‐mg caffeic acid) as a placebo, delivered by Unió Origen, SCCL (Spain) and (b) prebiotic supplementation based on FOS and inulin (FibroPur Inulin FOS) or maltodextrin as a placebo in powder format manufactured and delivered by Bonusan Besloten Vennootschap (BV) (the Netherlands). The subjects were randomly assigned to three groups: ROO and maltodextrin placebo as a control group (ROO group), EVOO rich in phenolic compounds and maltodextrin placebo (EVOO group) and EVOO rich in phenolic compounds and prebiotic supplementation (EVOO + PREB group). The dose was 30 mL/day of ROO or EVOO rich in phenolic compounds used as a dressing and 7.5 g/day of prebiotic supplementation or maltodextrin placebo diluted in a glass of water during or just after eating. For cooking, the volunteers used their usual oil. The placebo and the intervention products presented the same format and appearance to ensure the double‐blind intervention. Figure S1 show the FOOP‐Sarc study design.

All the volunteers received nutritional and physical activity recommendations, which the format was previously developed through a co‐creation process with a subsample of volunteers and study researchers [17]. The nutritional recommendations, called NFOC‐diet, were based on the Dietary Approaches to Stop Hypertension (DASH diet) [18], as it meets the specific requirements of people with sarcopenia. Also, NFOC‐diet is rich in protein (in particular, leucine [19]), vitamin D, polyunsaturated acids, phosphorus and iron [20, 21]. The physical activity recommendations are 150 min/week of moderate‐to‐vigorous physical activity, with at least two sessions of motor strength [16].

Seven visits took place during the intervention: an initial screening visit (V0), a baseline visit (V1), telephone visits (V2, V3 and V4), a final study visit at 12 weeks of intervention (V5) and a 12‐week follow‐up visit after intervention cessation (V6). All the study visits took place at the Centre Mèdic Quirúrgic (CMQ) of Reus. At visits V0, V1, V5 and V6, venous blood samples were collected in a sterile setting. At visits V1, V5 and V6, urine and faeces samples were collected. Biological samples were stored in the Biobanc of the Institut d'Investigació Sanitaria Pere Virgili (biobanc.reus@iispv.cat), blood samples at −80°C, whereas urine and faeces samples at −20°C.

The product's adherence was assessed by the bottles of olive oil and the jars of maltodextrin placebo/prebiotic returned at the end of the intervention, considering consumption of > 70% to be an acceptable level of adherence. Adverse effects were asked about at each visit throughout the study.

The study participants were randomly divided into three groups. The randomisation plan was generated using a website (www.randomization.com; accessed on 23 November 2022 at 10:46 AM CET) with 1:1 allocation using a random block. Participants, researchers and the statistician remained blinded to the type of product administered throughout the study. The study was approved by the Ethics Committee for Research with Medicines (Comité de Ética de Investigación con medicamentos—CEIm) (033/2022), and the protocol was registered on ClinicalTrials.gov (NCT05485402). Also, volunteers signed written informed consent before the initial visit. The present study was conducted following the Helsinki Declaration and Good Clinical Practice Guidelines of the International Conference of Harmonization (GCP ICH) and was reported as CONSORT criteria.

### 2.2 Study Population

Volunteers were recruited through news in the newspapers, primary care centres and media and social networks from the Universitat Rovira i Virgili and the research group. They were contacted by email and telephone. The inclusion criteria were as follows: (a) men and women of ≥ 60 and ≤ 80 years of age and (b) at least one of the sarcopenia parameters based on EWGSOP1: low muscle strength based on grip dynamometry (men < 30 kg; women < 20 kg), low skeletal muscle mass index (SMI) based on bioimpedance analysis (BIA) (men < 8.87 kg/m^2^; women < 6.42 kg/m^2^) and/or low physical performance based on 4‐m gait speed (≤ 0.8 m/s) [6]. For the FOOP‐Sarc Project, the EWGSOP1 cut‐off points were chosen as they are wider than those of the EWGSOP2 consensus, thus increasing the range of volunteer inclusion. However, the EWGSOP2 sarcopenia classification (probable sarcopenia, sarcopenia and severe sarcopenia) was used.

The exclusion criteria were as follows: (a) type 1 or type 2 diabetes; (b) anaemia (haemoglobin ≤ 13 g/dL in men and ≤ 12 g/dL in women); (c) intestinal malabsorption diseases; (d) fructose and/or sucrose intolerance; (e) malnutrition (albumin < 3.5 g/dL); (f) renal diseases; (g) chronic alcoholism; (h) current or past participation in a clinical trial or consumption of a research product in the 30 days before inclusion in the study; (i) institutionalised older adults; and (j) failure to follow the study guidelines. Additionally, the volunteers who did not meet all the inclusion criteria were excluded. It is necessary to have signed the informed consent before the initial visit.

### 2.3 Outcome Measures

#### 2.3.1 Muscle Mass by BIA and Other Anthropometric Parameters

Anthropometric and vascular parameters were measured at V0, V1, V5 and V6. Body composition was measured by a segmental multifrequency body composition analyser (TANITA MC‐780MA; Tanita Corp., Tokyo, Japan). Muscle mass expressed as the change in SMI (kg/m^2^) was assessed as the primary outcome between baseline (V1) and end‐of‐intervention (V5). Other muscle mass parameters were the SM (kg), appendicular skeletal muscle mass (ASM) (kg), appendicular skeletal muscle mass index (ASMI) (kg/m^2^) as quantity of muscle mass and phase angle (°) as quality of muscle mass. Moreover, height was assessed using a wall‐mounted stadiometer (Tanita Leicester Portable; Tanita Corp., Barcelona, Spain), and the waist circumference (WC) was measured using a 150‐cm anthropometric steel measuring tape (McAuley PA, 2014).

#### 2.3.2 Muscle Strength and Physical Performance

Muscle strength based on handgrip strength was measured in the dominant arm using a hydraulic handheld dynamometer (JAMAR Plus+ Dynamometer; Performance Health Supply Inc., Cedarburg). The measurement was performed twice, and the average of the two values was calculated. Additionally, muscle physical performance was measured twice by gait speed on the 4 m, and the average of the two values was calculated [1].

#### 2.3.3 Ultrasound and Isokinetic Assessment

Muscle mass was also assessed based on dominant upper leg ultrasound [22] and isokinetic assessment [23] explained specifically previously [24].

Ultrasound assessment was conducted by VINNO 5 (Vinno [Suzhou] Co. Ltd., China) at HAR‐mode with the musculoskeletal (MSK) superficial preset at a frequency of 10 MHz with a linear transducer (Vinno [Suzhou] Co. Ltd., China) at V1, V5 and V6. The measurement point was between the anterior super iliac spine and the proximal end of the patella at 30% proximal of the superior border of the patella, with the transducer placed perpendicular to the length of the leg in the quadriceps and rectus femoris measures, and with the transducer parallel to the long axis for pennation angle measure [24]. All measures were carried out three times, and the mean was calculated for analysis. The ultrasound muscle quantity parameters assessed from the quadriceps were subcutaneous adipose tissue thickness (cm) and quadriceps MT (cm) [8]. Also, the ultrasound muscle quantity parameters assessed from the rectus femoris were MT (Y axis) (cm) and CSA (cm^2^) [8]. Additionally, another parameter assessed was the pennation angle (°).

The isokinetic assessment was carried out with an isokinetic dynamometer (Biodex System 4; Biodex Medical Systems, New York, USA) at V1 and V5. The parameters assessed at an angular velocity of 180° s^−1^ and 240° s^−1^ in leg extension and flexion were maximum peak torque (Nm), maximum total work (J) and mean power (W) [25]. Also, the ratio between the mean power by isokinetic and the rectus femoris CSA by ultrasound reported information about muscle quality, especially muscle function [26].

#### 2.3.4 Other Outcomes

Methodology for assessing vascular, frailty, biochemical parameters, adherence to nutritional and physical activity recommendations and quality of life is detailed in the Supporting Information.

### 2.4 Sample Size

The sample size was calculated in GRANMO software (https://www.imim.cat/ofertadeserveis/software‐public/granmo/). Accepting an alpha risk of 0.05 and a beta risk of 0.2 in a two‐sided test, 13 subjects are necessary for each intervention arm to detect a minimum statistically significant difference of 1.15 kg/m^2^ in SMI by BIA between two groups, assuming there are three groups and a standard deviation of 0.78 kg/m^2^. It has been anticipated a dropout rate of 20%. Finally, the total sample size of the FOOP‐Sarc Project will be 39 subjects.

### 2.5 Statistical Analysis

The parametricity of the variables was examined, and a logarithmic transformation was performed if required. Differences in baseline characteristics among groups were assessed by the ANOVA test or χ^2^ expressed as mean ± standard deviation (SD) or percentages. Data imputation was performed by multiple regression analyses. A general linear model was used to assess the trend of values from baseline to end‐of‐intervention and to 12‐week follow‐up (after the cessation of the intervention), and data were expressed as mean ± standard error (SE). Differences among treatments were assessed by an ANCOVA test adjusted by age, sex, baseline values and changes in physical activity in parametric data. Data were expressed as mean or median and 95% confidence interval (CI). Wilcoxon, χ^2^ and Mann–Whitney tests were used to analyse intratreatment and intertreatment differences in nonparametric data. Analyses were made by intention‐to‐treat. Data presented are those in the whole population (all) and females, given the low number of males in the study.

## 3 Results

**FIGURE 1:**
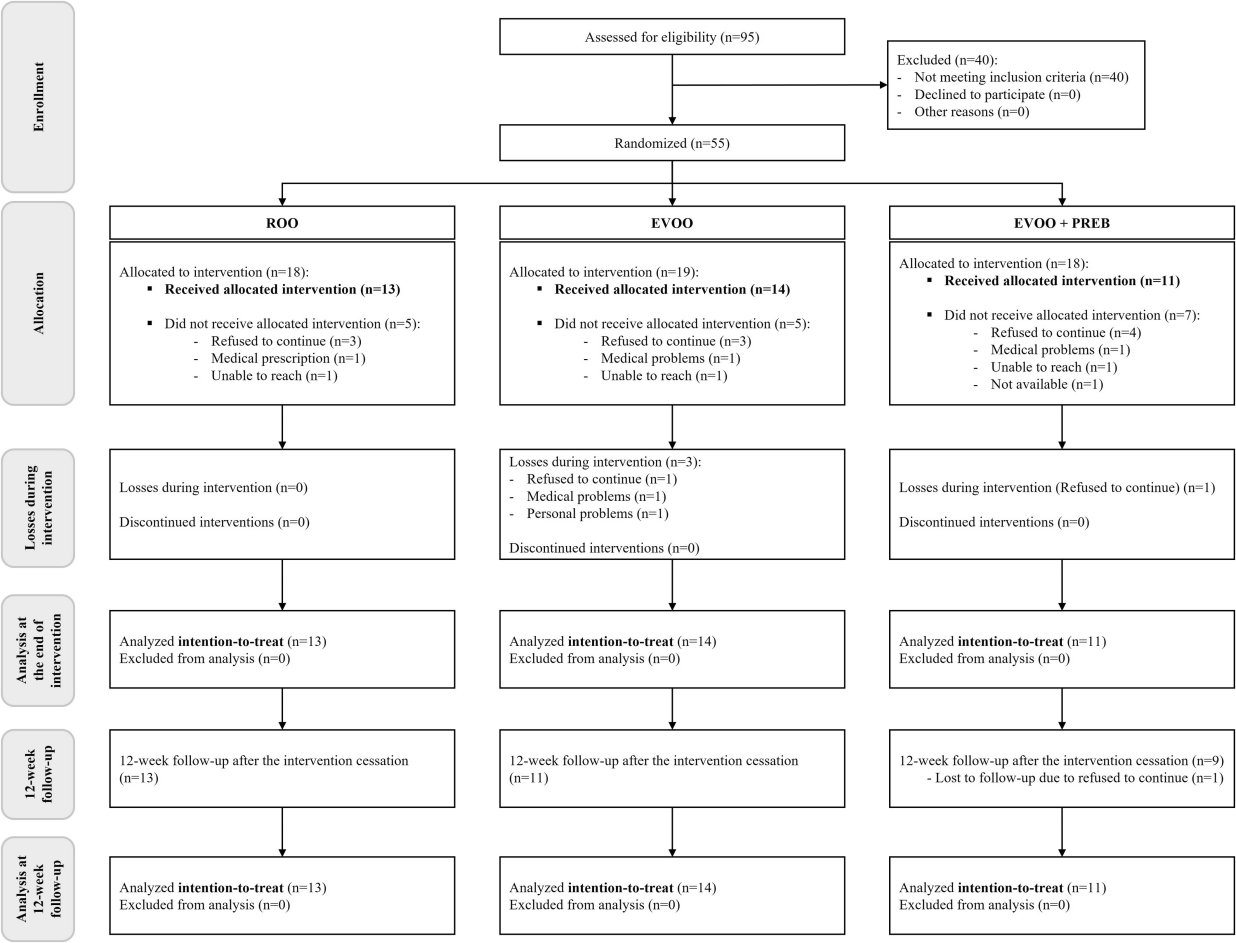
CONSORT flow diagram. The nutritional intervention was based on 30 mL/day of refined olive oil or extra virgin olive oil rich in phenolic compounds and 1 level tablespoon/day (7.5 g) of placebo (maltodextrin) or prebiotic (fructooligosaccharides and inulin). All intervention and control groups followed co‐created nutritional and physical activity recommendations. EVOO + PREB; extra virgin olive oil rich in phenolic compounds and prebiotic group; EVOO: extra virgin olive oil rich in phenolic compounds and maltodextrin placebo group; ROO: refined olive oil and maltodextrin placebo group.

From the 95 eligible volunteers, 40 volunteers were excluded due to not meeting inclusion criteria, and 55 volunteers were included and randomised (ROO *n* = 18; EVOO *n* = 19; EVOO + PREB *n* = 18). Of the 55 randomised volunteers, only 38 volunteers received the allocated intervention (ROO *n* = 13; EVOO *n* = 14; EVOO + PREB *n* = 11), whereas 17 volunteers did not receive the allocated intervention because they refused to continue (*n* = 10), had a medical prescription or medical problems (*n* = 3), unable to reach (*n* = 3) and being not available (*n* = 1). At the end, 34 volunteers finished the intervention, and four were dropouts during the intervention in EVOO (*n* = 3) and EVOO + PREB (*n* = 1) groups. Additionally, related to the 12‐week follow‐up, there was one dropout in the EVOO + PREB group. However, at the 12‐week follow‐up analysis, all 38 volunteers were analysed by intention‐to‐treat. All information is shown in the CONSORT flow diagram (Figure 1).

### 3.1 Characteristics of the Study Volunteers

The mean ± SD age of included volunteers was 69.6 ± 4.1 years, and 81.6% (*n* = 31/38) of the volunteers were women. Regarding sarcopenia parameters and based on EWGSOP1 cut‐off points, all of the volunteers had low muscle strength, and only six volunteers had also low physical performance. No volunteers had low muscle mass. According to the EWGSOP2 classification, all the volunteers had probable sarcopenia. No differences among treatments were observed at baseline in all population and females for smoking habits, SBP, DBP, weight, BMI, WC, fat and gait speed. However, grip strength was significantly lower in females of the EVOO group (*p* = 0.017). Table 1 shows the baseline characteristics of all participants and females. Additionally, regarding medication, none of the volunteers used glucocorticoids.

**TABLE 1:** Baseline characteristics of participants by intervention group.

Variable	All	EVOO + PREB (*n* = 11)	EVOO (*n* = 14)	ROO (*n* = 13)	*p*
Females, % (*n* = 31)	81.6	90.9	85.7	69.2	0.367
Age, years	69.6 ± 4.1	68.8 ± 4.7	70.0 ± 4.3	69.9 ± 3.7	0.751
Females	69.3 ± 4.3	68.9 ± 4.9	69.7 ± 4.5	69.1 ± 3.5	0.894
Smoking habits, %^a^, ^b^					
Non‐smoker	71.8	81.8	64.3	69.2	
Females	71.1	80.0	66.7	66.7	
Smoker	2.3	0.0	0.0	7.7	
Females	3.7	0.0	0.0	11.1	
Ex‐smoker	25.7	18.2	35.7	23.1	
Females	25.2	20.0	33.3	22.2	
SBP, mmHg	130 ± 16.0	121 ± 14.8	135 ± 13.1	133 ± 17.4	0.061
Females	130 ± 17.3	121 ± 15.6	135 ± 13.6	132 ± 21.1	0.142
DPB, mmHg	76 ± 9.2	74 ± 10.9	77 ± 8.3	76 ± 9.0	0.663
Females	76 ± 9.7	74 ± 11.4	79 ± 7.7	74 ± 10.1	0.235
Weight, kg	67.7 ± 13.1	64.3 ± 5.9	68.3 ± 18.2	70.0 ± 11.9	0.572
Females	63.6 ± 8.3	64.4 ± 6.2	62.5 ± 10.0	64.2 ± 9.0	0.857
BMI, kg/m^2^	27.4 ± 4.2	26.7 ± 3.0	27.1 ± 5.9	28.2 ± 3.2	0.652
Females	26.6 ± 3.3	27.0 ± 3.0	25.6 ± 3.9	27.3 ± 3.1	0.503
Waist circumference, cm	94.2 ± 12.2	91.9 ± 10.1	92.0 ± 16.1	98.5 ± 9.1	0.304
Females	91.5 ± 10.1	92.4 ± 10.5	87.6 ± 10.6	95.6 ± 7.7	0.187
Fat, %	34.8 ± 6.8	36.1 ± 6.9	32.9 ± 8.1	35.5 ± 5.5	0.483
Females	36.1 ± 6.4	37.5 ± 5.4	33.2 ± 7.9	38.2 ± 4.3	0.164
Grip strength, kg	19.3 ± 4.5	18.0 ± 1.3	16.4 ± 5.6	19.4 ± 5.5	0.271
Females	18.1 ± 2.8	18.0 ± 1.4	14.7 ± 3.9	17.4 ± 1.8	**0.017**
Gait speed, m/s	1.03 ± 0.18	1.04 ± 0.14	0.95 ± 0.22	1.11 ± 0.13	0.065
Females	1.01 ± 0.20	1.03 ± 0.15	0.93 ± 0.23	1.11 ± 0.15	0.101

*Note:* Data expressed as mean ± SD, or percentages. *p* for ANOVA or χ^2^. Significant differences are depicted in **bold**.

Abbreviations: BMI, body mass index; DBP, diastolic blood pressure; EVOO, extra virgin olive oil rich in phenolic compounds and maltodextrin placebo group; EVOO + PREB, extra virgin olive oil rich in phenolic compounds and prebiotic supplementation group; ROO, refined olive oil and maltodextrin placebo group; SBP, systolic blood pressure.

^a^ Smoking habits all population (*p* = 0.558).

^b^ Smoking habits females (*p* = 0.549).

### 3.2 Anthropometric Parameters

Regarding anthropometric parameters, at the end‐of‐intervention, no significant changes were observed among the groups. Nevertheless, at 12‐week follow‐up, a significant increase was observed in weight and consequently in BMI in the EVOO + PREB group (*p* = 0.023 and *p* = 0.034, respectively) and EVOO group (*p* = 0.025 and *p* = 0.047, respectively) when compared with the ROO group in all population (Table S1). No significant changes were observed among treatments for the other anthropometric parameters (Table S1).

### 3.3 Muscle Mass Assessed by BIA, Muscle Strength, Physical Performance and Frailty

**FIGURE 2:**
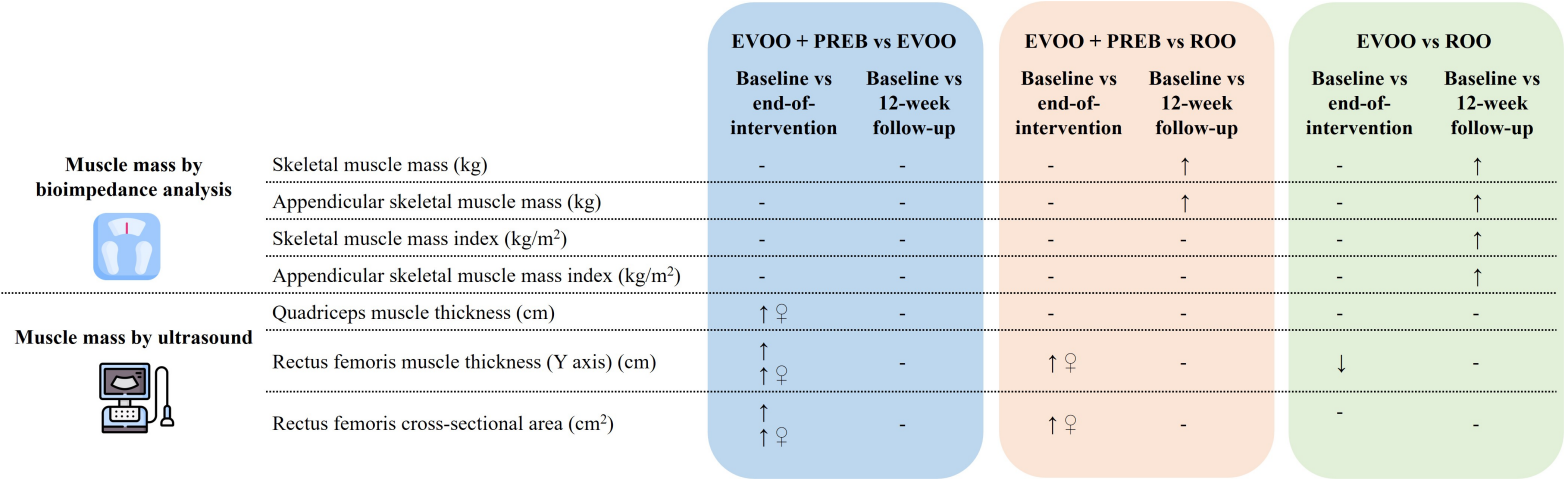
Muscle mass results assessed by BIA and ultrasound. EVOO + PREB; extra virgin olive oil rich in phenolic compounds and prebiotic group; EVOO: extra virgin olive oil rich in phenolic compounds and maltodextrin placebo group; ROO: refined olive oil and maltodextrin placebo group. Related to muscle mass assessed by BIA at 12‐week follow‐up, a significant increase in SM and ASM was shown in the EVOO + PREB group and EVOO group compared to the ROO group in all population. Additionally, at the 12‐week follow‐up, a significant increase was observed in SMI and ASMI in the EVOO group compared to the ROO group in all population. Concerning ultrasound parameters, for quadriceps MT at the end‐of‐intervention, a significant increase was shown in the EVOO + PREB group compared with the EVOO group in the female population. Related to the rectus femoris MT (Y axis) at the end‐of‐intervention, a significant increase was observed in the EVOO + PREB group compared to the EVOO group in all population and females. Also, at the end‐of‐intervention, a significant increase was shown for rectus femoris MT (Y axis) in the EVOO + PREB group compared to the ROO group in the female population. Moreover, at the end‐of‐intervention, rectus femoris MT (Y axis) significantly decreased in the EVOO group compared to the ROO group in all population. For rectus femoris CSA at the end‐of‐intervention, a significant increase was shown in the EVOO + PREB group compared with the EVOO group in all population and females. Also, at the end‐of‐intervention, a significant increase was shown in the EVOO + PREB group compared to the ROO group in females.

Related to muscle mass assessed by BIA at the end‐of‐intervention, no significant changes were observed among groups. But, at 12‐week follow‐up, a significant increase in SM and ASM was shown in the EVOO + PREB group (0.432 kg [0.33; 0.83], *p* = 0.035 and 0.353 kg [0.03; 0.68], *p* = 0.035, respectively) and EVOO group (0.430 kg [0.02; 0.84], *p* = 0.039 and 0.449 kg [0.12; 0.78], *p* = 0.010, respectively) compared to the ROO group in the all population (Table 2 and Figure 2). Also, increasing linear trends were observed for SM and ASM in the EVOO + PREB (*p* = 0.001 and *p* = 0.018, respectively) and EVOO (*p* = 0.009 and *p* = 0.014, respectively) groups for all population showing a sustained effect at 12‐week follow‐up (Table S2).

**TABLE 2:** Intertreatment changes in sarcopenia parameters.

Variable	Changes from baseline at the end‐of‐intervention						Changes from baseline at 12‐week follow‐up					
	EVOO + PREB vs. EVOO		EVOO + PREB vs. ROO		EVOO vs. ROO		EVOO + PREB vs. EVOO		EVOO + PREB vs. ROO		EVOO vs. ROO	
	Mean (95% CI)	*p*	Mean (95% CI)	*p*	Mean (95% CI)	*p*	Mean (95% CI)	*p*	Mean (95% CI)	*p*	Mean (95% CI)	*p*
Mass fat, %												
All	0.358 (−1.1; 1.8)	0.624	−0.307 (−1.7; 1.1)	0.666	−0.665 (−2.1; 0.76)	0.349	0.131 (−1.2; 1.4)	0.840	0.487 (−0.82; 1.8)	0.452	0.356 (−0.91; 1.6)	0.570
Female (*n* = 30)	−0.335 (−1.7; 1.0)	0.622	0.087 (−1.2; 1.4)	0.894	0.423 (−0.13; 0.14)	0.542	0.374 (−0.89; 1.6)	0.456	0.100 (−1.0; 1.2)	0.860	−0.274 (−1.6; 1.0)	0.668
Skeletal muscle mass, kg												
All	0.042 (−0.58; 0.68)	0.892	0.249 (−0.34; 0.84)	0.399	0.207 (−0.39; 0.80)	0.844	0.002 (−0.43; 0.43)	0.992	**0.432** **(0.33; 0.83)**	**0.035**	**0.430** **(0.02; 0.84)**	**0.039**
Female (*n* = 30)	0.185 (−0.42; 0.79)	0.532	0.127 (−0.48; 0.74)	0.671	−0.058 (−0.67; 0.56)	0.848	0.102 (−0.30; 0.51)	0.608	0.294 (−0.09; 0.68)	0.198	0.192 (−0.23; 0.61)	0.355
Skeletal muscle mass index, kg/m^2^												
All	0.031 (−0.24; 0.30)	0.816	0.041 (−0.22; 0.30)	0.756	0.009 (−0.25; 0.26)	0.940	−0.013 (−0.48; 0.45)	0.956	0.421 (−0.03; 0.87)	0.067	**0.434** **(0.001; 0.87)**	**0.050**
Female (*n* = 30)	0.104 (−0.18; 0.39)	0.455	−0.029 (−0.32; 0.26)	0.839	−0.133 (−0.42; 0.15)	0.346	0.012 (−0.56; 0.58)	0.967	0.421 (−0.13; 0.97)	0.126	0.409 (−0.17; 0.99)	0.159
Appendicular skeletal muscle mass, kg												
All	−0.145 (−0.69; 0.40)	0.595	0.168 (−0.35; 0.69)	0.517	0.313 (−0.21; 0.84)	0.232	−0.096 (−0.45; 0.26)	0.586	**0.353** **(0.03; 0.68)**	**0.035**	**0.449** **(0.12; 0.78)**	**0.010**
Female (*n* = 30)	0.038 (−0.32; 0.40)	0.829	−0.030 (−0.40; 0.34)	0.871	0.068 (−0.44; 0.30)	0.707	0.007 (−0.26; 0.27)	0.955	0.174 (−0.08; 0.43)	0.178	0.167 (−0.11; 0.45)	0.229
Appendicular skeletal muscle mass index, kg/m^2^												
All	−0.083 (−0.29; 0.13)	0.429	0.048 (−0.16; 0.25)	0.635	0.132 (−0.07; 0.33)	0.188	−0.059 (−0.20; 0.08)	0.404	0.122 (−0.01; 0.26)	0.076	**0.181** **(0.05; 0.31)**	**0.008**
Female (*n* = 30)	0.017 (−0.14; 0.18)	0.828	−0.026 (−0.19; 0.13)	0.742	−0.043 (−0.20; 0.12)	0.584	−0.025 (−0.14; 0.09)	0.667	0.052 (−0.06; 0.17)	0.364	0.077 (−0.04; 0.20)	0.206
Appendicular skeletal muscle mass, %^a^												
All	−0.010 (−1.0; 0.99)	0.985	0.418 (−0.57; 0.1.4)	0.393	0.427 (−0.56; 1.4)	0.382	−0.175 (−0.56; 0.21)	0.357	0.042 (−0.34; 0.43)	0.824	0.217 (−0.16; 0.60)	0.250
Female (*n* = 29)	0.436 (−0.29; 1.2)	0.226	0.172 (−0.56; 0.91)	0.633	−0.264 (−1.0; 0.49)	0.478	−0.232 (−0.69; 0.22)	0.602	−0.010 (−0.44; 0.43)	0.964	0.222 (−0.26; 0.70)	0.348
Angle phase, °												
All	0.344 (−0.20; 0.88)	0.205	0.300 (−0.25; 0.85)	0.275	−0.044 (−0.60; 0.52)	0.874	−0.071 (−0.76; 0.62)	0.835	0.096 (−0.61; 0.80)	0.783	0.168 (−0.60; 0.52)	0.639
Female (*n* = 30)	0.315 (−0.32; 0.95)	0.318	0.347 (−0.34; 1.0)	0.306	0.031 (−0.67; 0.73)	0.927	0.159 (−0.66; 0.98)	0.693	0.172 (−0.66; 1.0)	0.673	0.012 (−0.88; 0.90)	0.977
Hand grip strength (log), kg												
All	−0.039 (−0.09; 0.01)	0.137	−0.032 (−0.08; 0.02)	0.210	0.007 (−0.04; 0.06)	0.769	−0.013 (−0.08; 0.05)	0.680	−0.029 (−0.03; 0.09)	0.355	−0.016 (−0.08; 0.04)	0.591
Female (*n* = 30)	−0.025 (−0.07; 0.03)	0.338	−0.017 (−0.07; 0.03)	0.511	0.008 (−0.04; 0.06)	0.750	−0.014 (−0.09; 0.06)	0.712	−0.007 (−0.08; 0.06)	0.844	0.007 (−0.07; 0.08)	0.853
Gait speed, m/s												
All	−0.034 (−0.12; 0.05)	0.432	−0.003 (−0.09; 0.08)	0.950	0.031 (−0.06; 0.12)	0.469	0.004 (−0.11; 0.11)	0.942	0.037 (−0.07; 0.15)	0.507	0.036 (−0.08; 0.14)	0.546
Female (*n* = 30)	−0.003 (−0.09; 0.09)	0.948	−0.024 (−0.11; 0.07)	0.595	−0.021 (−0.12; 0.07)	0.653	−0.017 (−0.15; 0.11)	0.793	0.059 (−0.07; 0.18)	0.350	0.075 (−0.06; 0.21)	0.273

*Note:* Data expressed as mean (95% confidence interval, CI). ANCOVA Model adjusted by sex, age, baseline values and changes in physical activity. Significant differences are depicted in **bold**.

Abbreviations: EVOO, extra virgin olive oil rich in phenolic compounds and maltodextrin placebo group; EVOO + PREB, extra virgin olive oil rich in phenolic compounds and prebiotic supplementation group; ROO, refined olive oil and maltodextrin placebo group.

^a^ *n* = 37.

**FIGURE 3:**
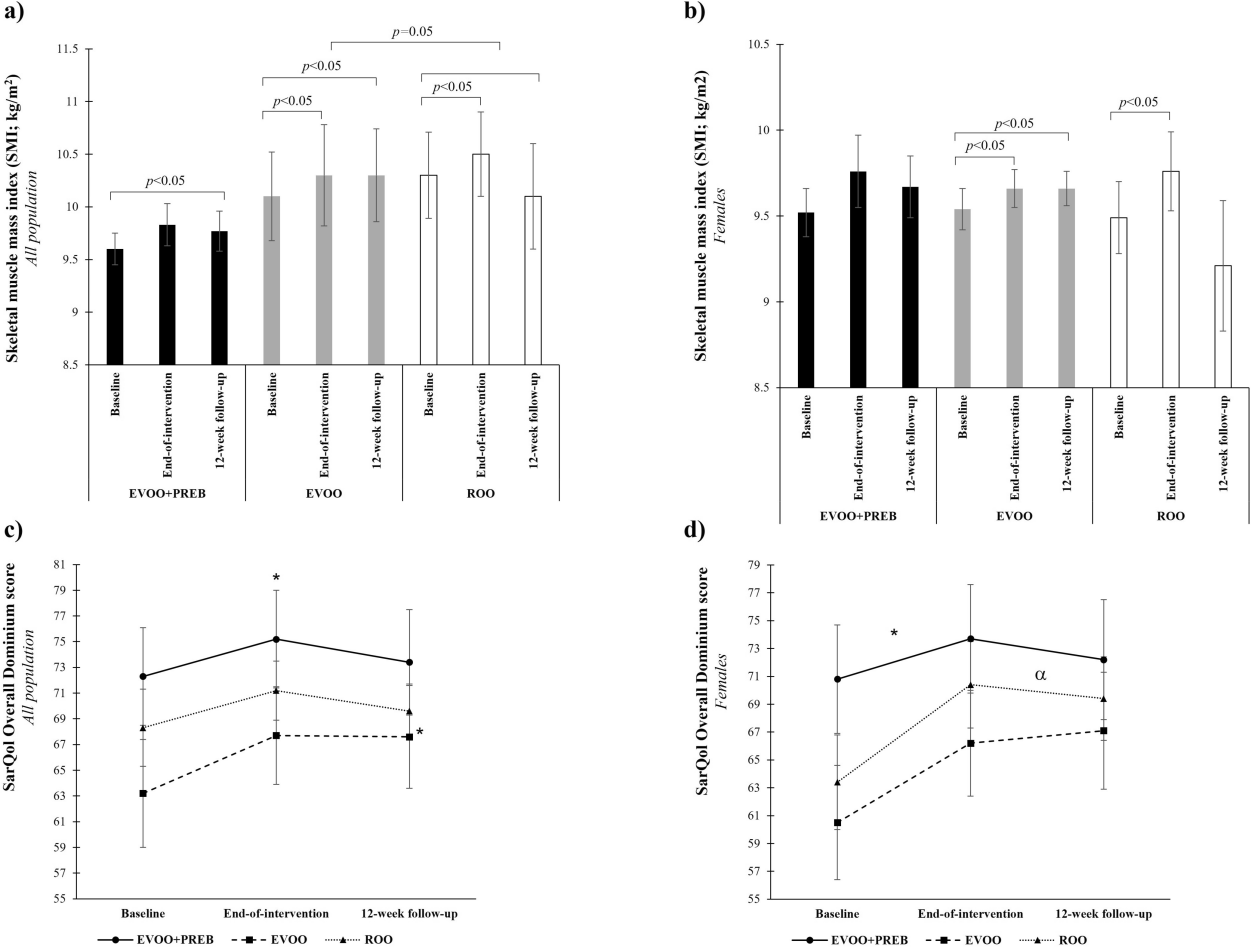
Changes in skeletal muscle mass index (kg/m^2^) and quality of life (SarQol questionnaire). (a) Changes in skeletal muscle mass index (kg/m^2^) in all population. (b) Changes in skeletal muscle mass index (kg/m^2^) in females. (c) Changes in quality of life (SarQol questionnaire) in all population. (d) Changes in quality of life (SarQol questionnaire) in females. EVOO + PREB; extra virgin olive oil rich in phenolic compounds and prebiotic group; EVOO: extra virgin olive oil rich in phenolic compounds and maltodextrin placebo group; ROO: refined olive oil and maltodextrin placebo group. *p < 0.05 versus baseline; α: *p* < 0.05 EVOO versus ROO intertreatment changes from baseline at 12‐week follow‐up. A *p*‐value < 0.05 is considered statistically significant.

Additionally, at the 12‐week follow‐up, a significant increase was observed in SMI and ASMI in the EVOO group compared to the ROO group (0.434 kg/m^2^ [0.001; 0.87], *p* = 0.050 and 0.181 kg/m^2^ [0.05; 0.31], *p* = 0.008, respectively) in the all population (Table 2 and Figures 2 and 3a,b). In this line, also increasing linear trends were observed for SMI and ASMI in the EVOO group for all population (*p* = 0.004 and *p* = 0.005, respectively) showing a sustained effect at 12‐week follow‐up (Table S2).

At the end‐of‐intervention and 12‐week follow‐up, no significant changes were observed among treatments for phase angle, handgrip strength, gait speed and frailty (Tables 2 and S2).

### 3.4 Ultrasound and Isokinetic Assessment

Concerning ultrasound parameters, for quadriceps MT at the end‐of‐intervention, a significant increase was shown in the EVOO + PREB group compared with the EVOO group (0.230 cm [0.008; 0.45], *p* = 0.044) in the female population. At 12‐week follow‐up, no significant changes were observed for quadriceps MT among groups (Table 3 and Figure 2).

**TABLE 3:** Intertreatment changes in muscle mass parameters assessed by ultrasound.

Variable	Changes from baseline at the end‐of‐intervention						Changes from baseline at 12‐week follow‐up					
	EVOO + PREB vs. EVOO		EVOO + PREB vs. ROO		EVOO vs. ROO		EVOO + PREB vs. EVOO		EVOO + PREB vs. ROO		EVOO vs. ROO	
	Mean (95% CI)	*p*	Mean (95% CI)	*p*	Mean (95% CI)	*p*	Mean (95% CI)	*p*	Mean (95% CI)	*p*	Mean (95% CI)	*p*
Subcutaneous adipose tissue thickness, cm												
All	0.058 (−0.06; 0.17)	0.314	0.031 (−0.08; 0.14)	0.576	−0.027 (−0.13; 0.08)	0.614	0.029 (−0.09; 0.14)	0.609	0.048 (−0.06; 0.16)	0.389	0.019 (−0.09; 0.13)	0.729
Female (*n* = 30)	0.047 (−0.09; 0.19)	0.502	0.053 (−0.08; 0.19)	0.420	0.006 (−0.13; 0.14)	0.925	−0.015 (−0.16; 0.13)	0.842	0.069 (−0.06; 0.20)	0.289	0.083 (−0.06; 0.23)	0.260
Quadriceps muscle thickness, cm												
All	0.209 (−0.06; 0.47)	0.119	0.048 (−0.21; 0.31)	0.712	−0.161 (−0.41; 0.09)	0.197	0.154 (−0.10; 0.41)	0.231	0.180 (−0.07; 0.44)	0.160	0.027 (−0.21; 0.26)	0.822
Female (*n* = 30)	**0.230** **(0.008; 0.45**)	**0.044**	0.181 (−0.05; 0.41)	0.119	−0.050 (−0.27; 0.18)	0.653	0.026 (−0.26; 0.31)	0.851	0.148 (−0.13; 0.43)	0.284	0.121 (−0.17; 0.41)	0.400
Rectus femoris muscle thickness (Y axis), cm												
All	**0.195** **(0.04; 0.35)**	**0.015**	0.034 (−0.12; 0.18)	0.648	**−0.161** **(−0.31; 0.01)**	**0.031**	0.171 (−0.005; 0.35)	0.057	0.145 (−0.03; 0.32)	0.098	−0.026 (−0.19; 0.14)	0.748
Female (*n* = 30)	**0.179** **(0.05; 0.31)**	**0.009**	**0.133** **(0.00; 0.27)**	**0.050**	−0.046 (−0.17; 0.08)	0.473	0.060 (−0.13; 0.25)	0.512	0.103 (−0.08; 0.28)	0.248	0.044 (−0.15; 0.23)	0.640
Rectus femoris cross‐sectional area, cm^2^												
All	**0.827** **(0.16; 1.5)**	**0.017**	0.235 (−0.42; 0.89)	0.472	−0.592 (−1.2; 0.04)	0.065	0.617 (−0.21; 1.4)	0.137	0.579 (−0.23; 1.4)	0.156	−0.038 (−0.81; 0.73)	0.920
Female (*n* = 30)	**0.569** **(−1.0; −0.08)**	**0.024**	**0.579** **(0.07; 1.1)**	**0.026**	0.011 (−0.49; 0.47)	0.964	−0.180 (−0.79; 0.43)	0.547	0.413 (−0.18; 1.0)	0.163	0.593 (−0.02; 1.2)	0.057
Pennation angle, °												
All	0.966 (−1.3; 3.2)	0.396	0.343 (−1.9; 2.5)	0.753	−0.623 (−2.7; 1.5)	0.549	−0.689 (−2.4; 1.0)	0.418	0.607 (−2.4; 1.0)	0.469	1.30 (−0.31; 2.9)	0.109
Female (*n* = 30)	0.982 (−1.4; 3.4)	0.408	0.671 (−1.7; 3.0)	0.561	−0.311 (−2.6; 2.0)	0.785	−0.166 (−2.3; 2.0)	0.875	0.503 (−1.4; 2.4)	0.583	0.670 (−1.5; 2.8)	0.526

*Note:* Data expressed as mean (95% confidence interval, CI). ANCOVA Model adjusted by sex, age, baseline values and changes in physical activity. Significant differences are depicted in **bold**.

Abbreviations: EVOO, extra virgin olive oil rich in phenolic compounds and maltodextrin placebo group; EVOO + PREB, extra virgin olive oil rich in phenolic compounds and prebiotic supplement group; ROO, refined olive oil and maltodextrin placebo group.

Related to the rectus femoris MT (Y axis) at the end‐of‐intervention, a significant increase was observed in the EVOO + PREB group compared to the EVOO group in all population (0.195 cm [0.04; 0.35], *p* = 0.015) and in females (0.179 cm [0.05; 0.31], *p* = 0.009). Also, at the end‐of‐intervention, a significant increase was shown for rectus femoris MT (Y axis) in the EVOO + PREB group compared to the ROO group in the female population (0.133 cm [0.00; 0.27], *p* = 0.050). Moreover, at the end‐of‐intervention, rectus femoris MT (Y axis) significantly decreased in the EVOO group compared to the ROO group (−0.161 cm [−0.31; 0.01], *p* = 0.031) in all population. At the 12‐week follow‐up, no significant changes were observed for rectus femoris MT (Y axis) among treatments (Table 3 and Figure 2).

For rectus femoris CSA at the end‐of‐intervention, a significant increase was shown in the EVOO + PREB group compared with the EVOO group in all population (0.827 cm^2^ [0.16; 1.5], *p* = 0.017) and in females (0.569 cm^2^ [−1.0; −0.08], *p* = 0.024). Also, at the end‐of‐intervention, a significant increase in the EVOO + PREB group compared to the ROO group in females (0.579 cm^2^ [0.07; 1.1], *p* = 0.026) was observed. At 12‐week follow‐up, no significant changes were observed for rectus femoris CSA among groups (Table 3 and Figure 2). In this line, a significant increasing quadratic trend was observed in the EVOO + PREB group in females (*p* = 0.038), showing an increase at the end‐of‐intervention but without a sustained effect over time (Table S3).

At the end‐of‐intervention and 12‐week follow‐up, no significant changes were observed among groups for the other parameters measured by ultrasound (Table 3 and Figure 2).

Regarding isokinetic dynamometer parameters at the end‐of‐intervention, for total work for extension at 180° s^−1^, a significant increase occurred in all groups. However, the EVOO + PREB group increased less than the EVOO group in all population (−0.058 J [−0.11; −0.04], *p* = 0.035) and females (−0.063 J [−0.12; 0.01], *p* = 0.022) (Table S4). Also, similar results were observed in females at 240° s^−1^ (−5.57 J [−10; −0.88], *p* = 0.022) at the end‐of‐intervention (Table S5). No significant changes were observed among treatments for the other isokinetic parameters (Tables S4 and S5).

### 3.5 Vascular Parameters

Concerning vascular parameters, at the end‐of‐intervention, no significant changes were observed among groups. At the 12‐week follow‐up, a significantly higher DBP was observed for the EVOO + PREB group compared to the EVOO group for all population and females (4.95 mmHg [−0.39; 1.5], *p* = 0.034 and 6.62 mmHg [−1.5; 12], *p* = 0.013, respectively) (Table S1), although the DBP levels were optimal. Besides, DBP for the EVOO group was significantly lower than the ROO group for the female population (−6.7 mmHg [−12; −1.5], *p* = 0.014) (Table S1). No significant changes were observed among treatments for vascular parameters (Table S1).

### 3.6 Biochemical Parameters

For IL‐6 at the end‐of‐intervention, no significant changes were observed among groups. However, at 12‐week follow‐up, a significant increase was shown in the EVOO + PREB group compared to the ROO group in all population (1.12 pg/mL [0.08; 2.1], *p* = 0.035) (Table S6).

For creatinine at the end‐of‐intervention, a significant increase was shown in the EVOO group compared to the ROO group in the all population (0.048 mg/dL [0.001; 0.10], *p* = 0.048). At 12‐week follow‐up, no significant changes were observed among groups (Table S6).

For cystatin C, at the end‐of‐intervention, no changes were observed among groups. However, at the 12‐week follow‐up, a significant increase was shown in the EVOO + PREB group compared to the ROO group in all population (0.062 mg/L [0.01; 0.1], *p* = 0.027) and females (0.064 mg/L [0.004; 0.1], *p* = 0.038) (Table S6).

For creatine kinase at the end‐of‐intervention, a significant decrease was observed in the EVOO + PREB group compared to the EVOO group in all population (−0.162 UI/L [−0.30; −0.02], *p* = 0.023) and females (−0.161 UI/L [−0.31; −0.01], *p* = 0.032). At 12‐week follow‐up, no significant changes were observed among groups (Table S6).

At the end‐of‐intervention and 12‐week follow‐up, no significant changes were observed among groups for the other biochemical parameters (Tables S6 and S7) or for the myostatin and follistatin parameters (Table S8).

### 3.7 Adherence to the Intervention, Tolerance and Adverse Effects of the Product, and Adherence to Nutritional and Physical Activity Recommendations

The adherence of the different intervention groups was (1) ROO group (ROO 67.16% and maltodextrin placebo 73.08%), (2) EVOO group (EVOO 79.08% and maltodextrin placebo 70.51%) and (3) EVOO + PREB group (EVOO 72.39% and prebiotic 84.22%), considering a consumption of > 70% to be an acceptable level of product compliance. No adverse events were reported, and all products were well tolerated.

Concerning dietary intake at the end‐of‐intervention and 12‐week follow‐up, no significant changes were observed among groups (Tables S9 and S10). Regarding physical activity at the end‐of‐intervention, no significant changes were observed among groups (Table S11). However, at 12‐week follow‐up, values for physical activity decreased significantly in the EVOO + PREB group compared to the EVOO group in females (−0.339 METs/week [−0.001; 0.68], *p* = 0.049) (Table S11) with a significant decreasing linear trend in females (*p* = 0.038) (Table S12).

### 3.8 Quality of Life of Individuals With Sarcopenia

Regarding the SarQol questionnaire at the end‐of‐intervention, no significant changes were observed among groups. For the overall domain at 12‐week follow‐up, a significant increase was observed in the EVOO group compared to the ROO group in females (6.48 score [0.52; 12], *p* = 0.034) (Table 4 and Figure 3c,d). Also, a significant increasing linear trend was observed in the EVOO group in females (*p* = 0.007) showing a sustained effect over time (Table S13).

**TABLE 4:** Intertreatment changes in quality of life (SarQol).

Variable	Changes from baseline at the end‐of‐intervention						Changes from baseline at 12‐week follow‐up					
	EVOO + PREB vs. EVOO		EVOO + PREB vs. ROO		EVOO vs. ROO		EVOO + PREB vs. EVOO		EVOO + PREB vs. ROO		EVOO vs. ROO	
	Mean (95% CI)	*p*	Mean (95% CI)	*p*	Mean (95% CI)	*p*	Mean (95% CI)	*p*	Mean (95% CI)	*p*	Mean (95% CI)	*p*
*Dominium 1 score*												
All	2.43 (−5.0; 9.9)	0.509	1.95 (−5.5; 9.4)	0.598	−0.483 (−7.4; 6.4)	0.888	−0.57 (−6.4; 6.3)	0.986	3.44 (−3.0; 9.9)	0.286	3.50 (−2.4; 9.4)	0.239
Female (*n* = 31)	3.84 (−4.8; 12)	0.369	2.34 (−6.4; 11)	0.588	−1.50 (−0.56; 0.69)	0.719	1.44 (−8.6; 5.7)	0.680	4.44 (−2.5; 11)	0.200	5.88 (−1.3; 13)	0.102
*Dominium 2 score*												
All	−0.663 (−8.8; 7.5)	0.870	−2.95 (−11; 5.2)	0.467	−2.28 (−9.9; 5.4)	0.547	−2.04 (−12; 7.9)	0.678	−0.436 (−10; 9.6)	0.930	1.61 (−7.6; 11)	0.725
Female (*n* = 31)	−1.78 (−10; 6.8)	0.673	1.84 (−11; 6.9)	0.670	−0.059 (−8.6; 8.5)	0.989	−8.34 (−17; 0.51)	0.064	1.64 (−7.0; 10)	0.698	**9.98 (0.99; 19)**	**0.031**
*Dominium 3 score*												
All	−0.131 (−5.8; 5.6)	0.963	−0.918 (−6.7; 4.9)	0.750	−0.787 (−6.1; 4.5)	0.764	0.907 (−6.4; 8.3)	0.803	3.10 (−4.5; 11)	0.412	2.19 (−4.6; 9.0)	0.518
Female (*n* = 31)	−0.439 (−7.0; 6.2)	0.892	−0.142 (−8.2; 5.3)	0.668	−0.984 (−7.4; 5.4)	0.754	−1.80 (−10; 6.9)	0.674	2.55 (−5.9; 11)	0.540	4.35 (−4.2; 13)	0.307
*Dominium 4 score*												
All	−0.462 (−6.8; 5.9)	0.883	0.105 (−6.2; 6.4)	0.973	0.567 (−5.3; 6.5)	0.846	−4.40 (−11; 2.3)	0.192	−4.23 (−11; 2.5)	0.213	0.174 (−6.0; 6.4)	0.955
Female (*n* = 31)	−1.42 (−8.5; 5.7)	0.685	0.709 (−6.5; 7.9)	0.841	2.13 (−4.9; 9.1)	0.537	**−8.37 (−15; −1.4)**	**0.020**	−5.21 (−12; 1.5)	0.124	3.16 (−3.8; 10)	0.358
*Dominium 5 score*												
All	7.29 (−0.98; 15)	0.082	4.50 (−3.5; 12)	0.263	−2.79 (−10; 4.8)	0.460	1.47 (−7.7; 11)	0.745	3.82 (−5.2; 13)	0.393	2.35 (−6.0; 11)	0.572
Female (*n* = 31)	5.73 (−3.6; 15)	0.217	5.00 (−4.1; 14)	0.268	−0.731 (−10; 8.6)	0.873	−2.73 (−13; 7.4)	0.583	5.48 (−4.1;15)	0.250	8.21 (−2.0;18)	0.110
*Dominium 6 score*												
All	−1.39 (−16;14)	0.852	5.04 (−9.5; 20)	0.486	6.44 (−8.3; 21)	0.381	−9.64 (−23; 4.2)	0.166	−10.5 (−24; 3.0)	0.123	−0.836 (−14; 13)	0.901
Female (*n* = 31)	0.206 (−16; 17)	0.980	5.18 (−11; 22)	0.528	4.96 (−13; 23)	0.579	−12.3 (−29; 4.0)	0.133	−9.78 (−25; 5.4)	0.198	2.54 (−15; 20)	0.771
*Dominium 7 score*												
All	−1.12 (−7.7; 5.5)	0.730	−1.46 (−8.1; 5.2)	0.656	−0.340 (−6.6; 5.9)	0.913	**−7.77 (−14; −1.2**)	**0.021**	−4.36 (−11; 2.3)	0.190	3.40 (−2.8; 9.6)	0.269
Female (*n* = 31)	−0.763 (−7.6; 6.1)	0.820	−0.984 (−8.1; 6.1)	0.779	−0.221 (−7.1; 6.7)	0.948	**−8.36 (−14; −2.3**)	**0.009**	−1.73 (−7.8; 4.3)	0.560	**6.62 (0.41; 13)**	**0.038**
*Overall Dominium score*												
All	0.177 (−6.3; 6.7)	0.956	1.19 (−5.2; 7.6)	0.709	1.01 (−5.0; 7.0)	0.734	−1.86 (−8.0; 4.2)	0.540	−0.189 (−6.3; 5.9)	0.950	1.67 (−3.9; 7.3)	0.549
Female (*n* = 31)	−0.519 (−8.0; 7.0)	0.889	1.35 (−6.1; 8.8)	0.712	1.87 (−5.6; 9.3)	0.609	−5.82 (−12; 0.12)	0.054	0.659 (−5.0; 6.3)	0.813	**6.48 (0.52; 12)**	**0.034**

*Note:* Data expressed as mean (95% confidence interval, CI). ANCOVA Model adjusted by sex, age, baseline values and changes in physical activity. Significant differences are depicted in **bold**.

Abbreviations: EVOO, extra virgin olive oil rich in phenolic compounds and maltodextrin placebo group; EVOO + PREB, extra virgin olive oil rich in phenolic compounds and prebiotic supplement group; ROO, refined olive oil and maltodextrin placebo group.

For domain 2 (Locomotion/Mobility) at 12‐week follow‐up, a significant increase was shown in the EVOO group compared to the ROO group in the female population (9.98 score [0.99; 19], *p* = 0.031). About domain 4 (functionality) at 12‐week follow‐up, a significant decrease was observed in the EVOO + PREB group compared to the EVOO group in females (−8.37 score [−15; 1.4], *p* = 0.020) (Table 4). Also, a significant increasing linear trend was observed in the EVOO group in females (*p* = 0.045), showing a sustained effect over time (Table S13). Also, for domain 7 (fears) at 12‐week follow‐up, a significant decrease was shown in the EVOO + PREB group compared to the EVOO group in all population (−7.77 score [−14; 1.2], *p* = 0.021) and females (−8.36 score [−14; 2.3], *p* = 0.009). Moreover, at the 12‐week follow‐up, a significant increase was observed for domain 7 in the EVOO group compared to the ROO group in females (6.62 score [0.41; 13], *p* = 0.038).

At the 12‐week follow‐up, no changes were observed among groups for the other domains (Table 4). The changes observed in domain 4 (functionality) and domain 7 (fears) were due to these variables increasing in the EVOO group compared to the EVOO + PREB group (Table S13).

## 4 Discussion

The present study confirmed that muscle parameters assessed by ultrasound, the EVOO + PREB intervention led to significant improvements at the end‐of‐intervention. Specifically, in females, an increase in quadriceps MT was observed compared to the EVOO group, while enhancements in rectus femoris MT (Y axis) and CSA were found in females compared to the ROO group and in all population and females compared to the EVOO group. At the 12‐week follow‐up, both the EVOO and EVOO + PREB groups showed significant increases in SM and ASM assessed by BIA in all population when compared to the ROO group. Furthermore, EVOO significantly improved SMI and ASMI, reinforcing its sustained effect over time in all population.

In general, muscle mass is estimated to decline by 1%–2% annually from 35 years, accelerating to ≥ 3% after 60 years [27, 28]. In young adults, the median (interquartile range) of the rectus femoris CSA is approximately 6.1 cm^2^ (5.1; 7.3) [29]. A person with 6.1 cm^2^ at age 35 would reach 3.16 cm^2^ at age 65, with a loss of 0.52 cm^2^ between 60 and 65 years. Therefore, the increase of 0.569–0.827 cm^2^ with the EVOO + PREB intervention could counteract this decline. Also, the relevance of rectus femoris CSA assessed by ultrasound and phase angle assessed by BIA should not be underestimated, as they have been identified in previous research as prognostic markers for 12‐month mortality in patients with conditions like idiopathic pulmonary fibrosis [30]. The present study highlights the potential of targeted nutritional interventions to increase muscle mass in older adults, as shown by ultrasound and BIA. Morphological changes were detected by ultrasound at the end‐of‐intervention, while total muscle mass increased at 12‐week follow‐up observed by BIA. However, no statistically significant changes were observed among groups in muscle strength and physical performance. This discrepancy is likely due to a delay in neuromuscular adaptations, which are essential for observing improvements in strength and physical performance [31]. Increases in muscle size and morphological changes can occur relatively quickly, but translating these changes into functional gains such as muscle strength and physical performance requires a longer time [31]. Physical exercise is one of the most effective interventions for accelerating neuromuscular adaptation and thereby enhancing strength and functional performance [31].

Additionally, sarcopenia is also part of the frailty diagnostic criteria [1, 32]. One prospective cohort study conducted in Spain determined that higher EVOO consumption (30 mL/day), as in the present study, was associated with a reduction of frailty risk [33]. Besides, prebiotic supplementation could improve muscle mass by modulating gut microbiota, since sarcopenia has been associated with an imbalance in gut bacteria profile characterised by increased abundance of species such as *Desulfovibrio piger* and *Clostridium symbiosum* and a reduced diversity of butyrate‐producing bacteria, which are important for maintaining muscle function and sarcopenia parameters [34]. Prebiotics selectively stimulate the growth of beneficial bacteria, which increase short‐chain fatty acid (SCFA) production, particularly butyrate [35]. These SCFA help reduce systemic inflammation and improve muscle protein synthesis and mitochondrial function, thereby contributing to the preservation of muscle mass in older adults [35]. In this context, a 13‐week RCT from Spain using the same prebiotic as the present study with the same dose observed a reduction of frailty levels assessed by the frailty index, in particular handgrip strength, compared to placebo [14, 36]. Nevertheless, in the present study, no changes in frailty were observed.

Concerning isokinetic parameters, changes > 10% in isokinetic variables are generally considered functionally significant [37, 38]. In the EVOO group, at the end‐of‐intervention, in females, an increase of 11.0% and 21.3% was shown for total work for extension at 180° s^−1^ and 240° s^−1^, respectively, and an increase of 22.3% for the ratio of muscle function for extension at 180° s^−1^. These results were considered functionally significant and suggest a clinical impact.

Regarding quality of life, it improved in females from the EVOO group, with an increase of 6.48 points at the 12‐week follow‐up compared to the ROO group. This change indicates a positive trend and may still be meaningful, particularly given the relatively short duration of the intervention. However, the observed increase of 6.48 points falls slightly below the suggested threshold for a minimal clinically important difference, which is defined as a change of at least 7.35 points in the overall domain (on a 0–100 scale) [39]. Moreover, a cross‐sectional study classifies the overall score of the SarQol questionnaire into three risk areas: ≤ 60 points (high risk of sarcopenia), 61–85 points (moderate risk of sarcopenia) and ≥ 86 points (low risk of sarcopenia) [40]. Female participants in the EVOO group changed from the high‐risk category (60.5 ± 4.1 points at baseline) to the moderate‐risk category (66.2 ± 3.8 points at the end‐of‐intervention and 67.1 ± 4.2 points at 12‐week follow‐up) for sarcopenia throughout the study.

One of the main mechanisms through which EVOO may exert protective effects is by modulating oxidative stress and inflammation [11]. EVOO influences signalling pathways via Nrf‐2 activation and inhibits NF‐κB pathways, reducing oxidative damage and inflammation [11]. Moreover, EVOO reduced mitochondrial oxygen species in animal models through Sirt1 activation and PGC1‐α expression [11, 41]. Also, the anti‐inflammatory effect of EVOO counteracts inflammatory cytokines such as tumour necrosis factor (TNF‐α), interleukin (IL‐6) and interferon‐γ (IFN‐γ), which contribute to muscle loss [10, 11]. A systematic review and meta‐analysis of observational studies concluded that a lower intake of monounsaturated fatty acids (MUFAs) present in EVOO is associated with a higher risk of sarcopenia [42]. MUFAs preserve muscle mass and function by mitigating ROS‐induced atrophy [42]. Moreover, EVOO is one of the main components of the Mediterranean diet, and its anti‐inflammatory and antioxidant properties reduce fractures and sarcopenia by the gut‐bone and gut‐muscle axes [43]. These positive effects were attributed to phenolic compounds, especially hydroxytyrosol and tyrosol [11].

Concerning the relation between prebiotic supplementation and EVOO intake, a potential mechanistic explanation for this unclear interaction may involve modulation of the gut‐muscle axis through microbial metabolites. EVOO polyphenols, such as hydroxytyrosol, can favour beneficial bacteria and stimulate SCFA production, similarly to prebiotics [44, 45, 46]. Their combination might therefore enhance SCFA‐mediated effects on muscle metabolism and inflammation [45, 46]. However, some bacteria can metabolise both types of compounds, so their combination may not always produce purely additive effects. Competition for microbial metabolic pathways could modulate overall SCFA production and subsequent effects on muscle. Further studies are needed to determine whether this combination results in synergistic, additive or inhibitory effects on the gut‐muscle axis.

**FIGURE 4:**
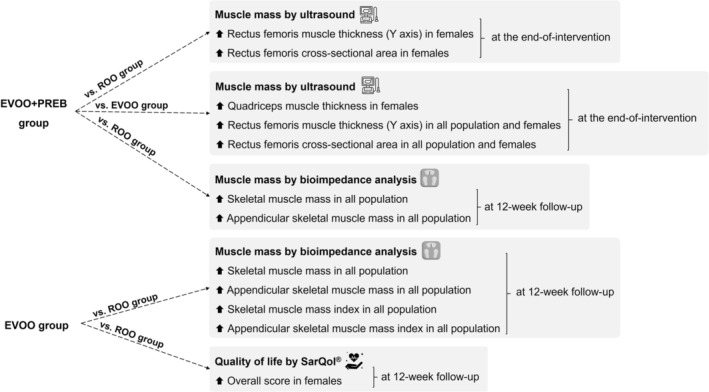
Discussion summary of both intervention groups' effects. EVOO + PREB; extra virgin olive oil rich in phenolic compounds and prebiotic group; EVOO: extra virgin olive oil rich in phenolic compounds and maltodextrin placebo group; BIA: bioimpedance analysis.

Thus, a product commonly used, such as EVOO, alone or in combination with a prebiotic, could represent a novel strategy to tackle sarcopenia and improve the quality of life of community‐dwelling older adults (Figure 4).

### 4.1 Limitations

This study had some limitations. Firstly, the small sample size—particularly the small number of males—may have influenced the results. Future studies should increase the sample size, maintaining the sex balance. Secondly, differences in adherence between the intervention groups (67%–84%) could interfere with the results, particularly in comparisons with ROO, where adherence to the oil was 67.16%. Thirdly, the potential synergy or inhibition between the prebiotic and the EVOO remains unclear. Future studies could investigate this aspect in addition to proposing a new arm in the intervention that only evaluates the effect of the prebiotic.

### 4.2 Conclusion

The consumption of EVOO rich in phenolic compounds in combination with prebiotic supplementation led to improvements in ultrasound parameters of the quadriceps and rectus femoris at the end‐of‐intervention. Moreover, at 12‐week follow‐up, EVOO—either alone or combined with a prebiotic formulation based on FOS and inulin—improved muscle mass assessed by BIA. Notably, at 12‐week follow‐up, EVOO intake was also associated with an improvement in overall quality of life. These findings suggest that EVOO rich in phenolic compounds may represent a promising nutritional strategy for supporting muscle health and quality of life in older adults. However, further studies are needed to confirm these results and to explore the potential effects of this intervention on additional sarcopenia‐related parameters.

## Funding

The authors acknowledge the 2021‐SGR‐00817 project from Agencia de Gestión de Ayudas Universitarias y de Investigación (AGAUR), Generalitat de Catalunya. This article in journal has been possible with the support of the Secretaria d‘Universitats i Recerca del Departament d‘Empresa i Coneixement de la Generalitat de Catalunya, the European Union (UE) and the European Social Fund (ESF) (2022 FI_B2 00011).

## Ethics Statement

The study was approved by the Ethics Committee for Research with Medicines (Comité de Ética de Investigación con medicamentos—CEIm) (033/2022) and the protocol was registered on ClinicalTrials.gov (NCT05485402; https://clinicaltrials.gov/study/NCT05485402; registration date: 03/08/2022). Volunteers signed written informed consent before the initial visit. The present study was conducted following the Helsinki Declaration and Good Clinical Practice Guidelines of the International Conference of Harmonization (GCP ICH) and was reported as CONSORT criteria.

## Conflicts of Interest

The authors declare no conflicts of interest.

## Supporting information


**Data S1:** Supporting information.

**Figure S1:** FOOP‐Sarc study design. EVOO + PREB; extra virgin olive oil rich in phenolic compounds and prebiotic group; EVOO: extra virgin olive oil rich in phenolic compounds and maltodextrin placebo group; ROO: refined olive oil and maltodextrin placebo group.

**Table S1:** Intertreatment changes in anthropometric and vascular parameters.
**Table S2:** Trends in sarcopenia parameters.
**Table S3:** Trends in muscle mass parameters assessed by ultrasound.
**Table S4:** Changes in muscle mass parameters assessed by isokinetic dynamometer at 180° s^−1^ at the end‐of‐intervention.
**Table S5:** Changes in muscle mass parameters assessed by isokinetic dynamometer at 240° s^−1^ at the end‐of‐intervention.
**Table S6:** Intertreatment changes in selected biochemical parameters.
**Table S7:** Changes in C‐reactive protein (mg/dL).
**Table S8:** Changes in myostatin and follistatin at the end‐of‐intervention.
**Table S9:** Energy, alcohol, fibre and selected nutrients and minerals at baseline, at the end‐of‐intervention and at 12‐week follow‐up in all population.
**Table S10:** Energy, alcohol, fibre and selected nutrients and minerals at baseline, at the end‐of‐intervention and at 12‐week follow‐up in the female population.
**Table S11:** Intertreatment changes in physical activity (METs/week).
**Table S12:** Trends in physical activity (METs/week, log) throughout the study.
**Table S13:** Trends in quality of life (SarQol) parameters.
