# Supplementary material for: Effects of Phenolic‐Rich Extra Virgin Olive Oil and Prebiotics on Sarcopenia in Older Adults: FOOP‐Sarc Project
Source: J Cachexia Sarcopenia Muscle. 2026 Mar 5;17(2):e70247. doi: 10.1002/jcsm.70247 (PMC12963666; doi:10.1002/jcsm.70247)
Supplement: Supplementary file 1 — Data S1: Supporting information. [file JCSM-17-e70247-s003.pdf]

Effects of Phenolic-Rich Extra Virgin Olive Oil and Prebiotics on Sarcopenia in Older Adults: FOOP-Sarc Project

SUPPLEMENTARY METHODS

Outcome measures

Vascular parameters

Systolic blood pressure (SBP), diastolic blood pressure (DBP), and pulse pressure (PP) were assessed after 2-5 minutes at rest using an automatic sphygmomanometer (OMRON-M6 Comfort (HEM-7360-E); Peroxfarma, Barcelona, Spain). Blood pressure was measured twice at 1-min intervals and the mean was calculated for analysis.

Frailty

Frailty was assessed by Fried criteria [1] at V1, V5, and V6 according to 5 domains: 1) unintentional weight loss, 2) weakness based on grip strength, 3) poor endurance and energy, 4) slowness, and 5) low physical activity level [1].

Biochemical parameters

All biochemical parameters were assessed at V1, V5, and V6. Serum vitamin D was assessed by standardized automated methods in an autoanalyzer (Beckman Coulter-Synchron, Galway, Ireland).

Inflammation biomarkers, such as serum high-sensitivity C-reactive protein (hsCRP) and IL-6 were measured by immunoturbidimetry in an autoanalyzer (Beckman Coulter-Synchron, Galway, Ireland). Serum uric acid was assessed by standardized enzymatic automated methods in an autoanalyzer (Beckman Coulter-Synchron, Galway, Ireland).

Regarding renal function, creatinine was measured by standardized methods in an autoanalyzer (Beckman Coulter-Synchron, Galway, Ireland), and cystatin C was measured by immunoturbidimetry in an autoanalyzer (Beckman Coulter-Synchron, Galway, Ireland) in 24 h urine samples.

Additionally, to asses muscle mass, wasting and turnover, myostatin, follistatin, and ratio myostatin/follistatin in serum samples were measured at V1 and V5 by Human Myostatin MSTN ELISA Kit and Human Follistatin

Like Protein 1 (FSTL1) ELISA Kit. Also, serum creatine kinase was assessed at V0, V1, V5, and V6 by standardized automated methods in an autoanalyzer (Beckman Coulter-Synchron, Galway, Ireland).

Adherence to nutritional and physical activity recommendations

The adherence to nutritional recommendations was assessed by a 3-day record at V1, V5, and V6, and a food frequency questionnaire (FFQ) at V2, V3, and V4. The mean daily intake of energy and nutrients was calculated using the 2020 CIQUAL food composition table [2]. Also, to assess the adherence to physical activity recommendations the International Physical Activity Questionnaire Adapted to Elderly (IPAQ-E) [3] was used at V1, V5, and V6.

Quality of life of individuals

To evaluate the quality of life of individuals with sarcopenia the SarQol® questionnaire was used at V1, V5, and V6 based on 7 domains [4]: D1) Physical and Mental health, D2) Locomotion/Mobility, D3) Body composition, D4) Functionality, D5) Activities of daily living, D6) Leisure activities, and D7) Fears [4,5]. To calculate each dominium and overall score, a pre-programmed Access database from the lead authors of the publication was used [4].

References

1. Fried LP, Tangen CM, Walston J, et al. Frailty in older adults: evidence for a phenotype. *J Gerontol A Biol Sci Med Sci*. 2001;56(3):M146-56. doi:10.1093/GERONA/56.3.M146
2. ANSES. CIQUAL food composition table 2020. Zenodo. 2020. Accessed July 22, 2024. https://doi.org/10.5281/zenodo.4770202
3. Rubio Castañeda FJ, Tomás Aznar C, Muro Baquero C. Validity, Reliability and Associated Factors of the International Physical Activity Questionnaire Adapted to Elderly (IPAQ-E). *Rev Esp Salud Publica*. 2017;91.
4. Beaudart C, Biver E, Reginster JY, et al. Validation of the SarQoL®, a specific health-related quality of life questionnaire for Sarcopenia. *J Cachexia Sarcopenia Muscle*. 2017;8(2):238-244. doi:10.1002/jcsm.12149
5. Montero-Errasquín B, Vaquero-Pinto N, Sánchez-Cadenas V, et al. Spanish translation, cultural adaptation and validation of the SarQoL®: a specific health-related quality of life questionnaire for sarcopenia. *BMC Musculoskelet Disord*. 2022;23(1). doi:10.1186/S12891-022-05125-Y
