# Supplementary figures and images for: Effects of Phenolic‐Rich Extra Virgin Olive Oil and Prebiotics on Sarcopenia in Older Adults: FOOP‐Sarc Project
Source: J Cachexia Sarcopenia Muscle. 2026 Mar 5;17(2):e70247. doi: 10.1002/jcsm.70247 (PMC12963666; doi:10.1002/jcsm.70247)

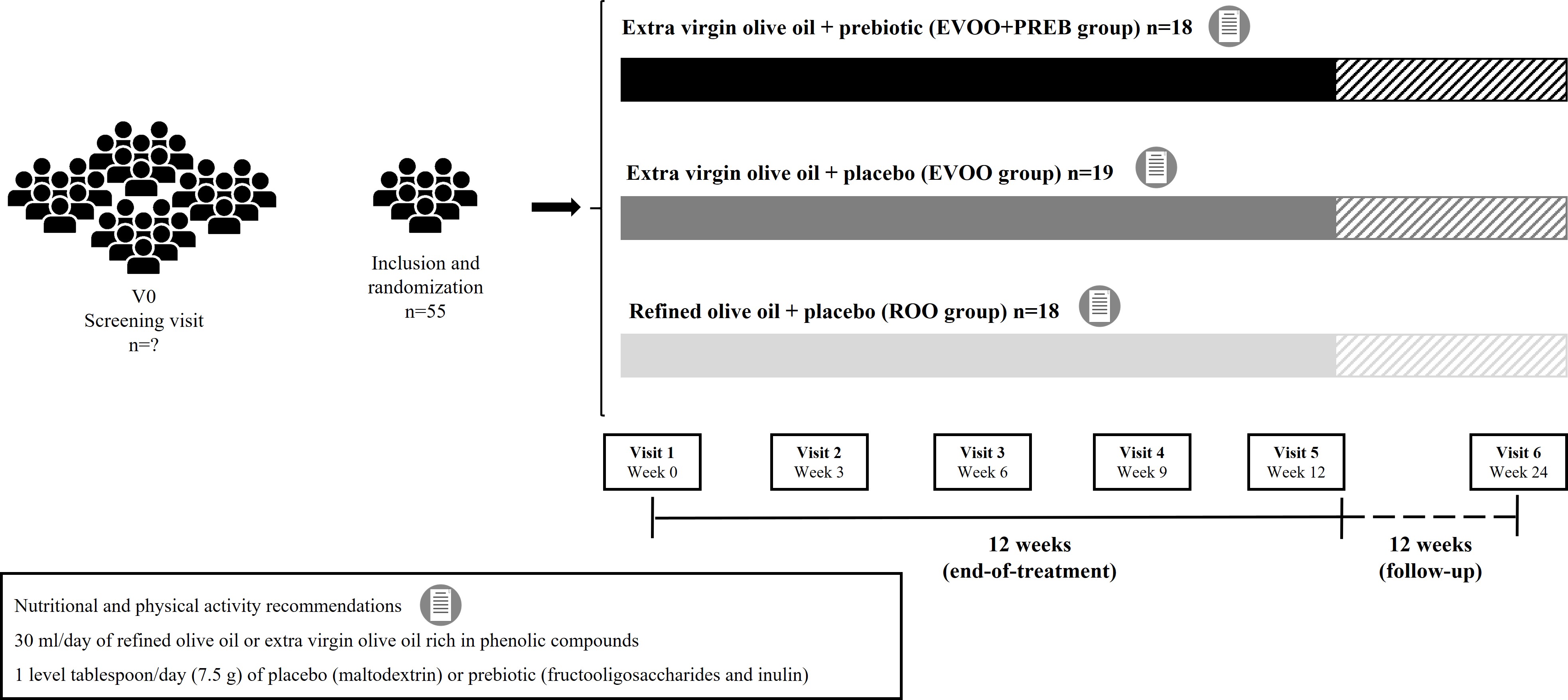

Supplement: Supplementary file 2 — Figure S1: FOOP‐Sarc study design. EVOO + PREB; extra virgin olive oil rich in phenolic compounds and prebiotic group; EVOO: extra virgin olive oil rich in phenolic compounds and maltodextrin placebo group; ROO: refined olive oil and maltodextrin placebo group. [file JCSM-17-e70247-s002.jpg]
